# Fetal alcohol exposure leads to abnormal olfactory bulb development and impaired odor discrimination in adult mice

**DOI:** 10.1186/1756-6606-4-29

**Published:** 2011-07-07

**Authors:** Katherine G Akers, Steven A Kushner, Ana T Leslie, Laura Clarke, Derek van der Kooy, Jason P Lerch, Paul W Frankland

**Affiliations:** 1 Neurosciences and Mental Health, Hospital for Sick Children, Toronto, Ontario, Canada; 2 Department of Psychiatry, Erasmus Medical Center, Rotterdam, The Netherlands; 3 Department of Molecular Genetics, University of Toronto, Toronto, Ontario, Canada; 4 Mouse Imaging Centre, Hospital for Sick Children, Toronto, Ontario, Canada; 5 Department of Medical Biophysics, University of Toronto, Toronto, Ontario, Canada; 6 Department of Physiology, University of Toronto, Toronto, Ontario, Canada; 7 Institute of Medical Science, University of Toronto, Toronto, Ontario, Canada

**Keywords:** fetal alcohol exposure, MRI, olfactory bulb, subependymal zone, odor discrimination, odor memory, neurospheres, neurogenesis

## Abstract

**Background:**

Children whose mothers consumed alcohol during pregnancy exhibit widespread brain abnormalities and a complex array of behavioral disturbances. Here, we used a mouse model of fetal alcohol exposure to investigate relationships between brain abnormalities and specific behavioral alterations during adulthood.

**Results:**

Mice drank a 10% ethanol solution throughout pregnancy. When fetal alcohol-exposed offspring reached adulthood, we used high resolution MRI to conduct a brain-wide screen for structural changes and found that the largest reduction in volume occurred in the olfactory bulbs. Next, we tested adult mice in an associative olfactory task and found that fetal alcohol exposure impaired discrimination between similar odors but left odor memory intact. Finally, we investigated olfactory bulb neurogenesis as a potential mechanism by performing an *in vitro *neurosphere assay, *in vivo *labeling of new cells using BrdU, and *in vivo *labeling of new cells using a transgenic reporter system. We found that fetal alcohol exposure decreased the number of neural precursor cells in the subependymal zone and the number of new cells in the olfactory bulbs during the first few postnatal weeks.

**Conclusions:**

Using a combination of techniques, including structural brain imaging, *in vitro *and *in vivo *cell detection methods, and behavioral testing, we found that fetal alcohol exposure results in smaller olfactory bulbs and impairments in odor discrimination that persist into adulthood. Furthermore, we found that these abnormalities in olfactory bulb structure and function may arise from deficits in the generation of new olfactory bulb neurons during early postnatal development.

## Background

Approximately 1% of children in the United States display symptoms of Fetal Alcohol Spectrum Disorder (FASD) [1], a classification encompassing the full range of subtle to severe birth defects resulting from the consumption of alcohol during pregnancy [2]. Although some symptoms are outwardly visible, such as stunted growth and craniofacial abnormalities, one of the most pronounced effects is damage to the developing brain. Neuroimaging studies reveal that children with FASD possess widespread brain abnormalities including reductions in the volume of the corpus callosum, cerebellum, basal ganglia, and hippocampus [3-5]. Presumably, these brain anomalies contribute to the complex array of behavioral disturbances observed in children with FASD including hyperactivity, attention deficits, impaired learning and memory, social problems, anxiety, and depression [5-9].

The high incidence of FASD warrants further study into its biological basis and behavioral manifestations, but it is often difficult to disentangle the links between gestational alcohol exposure and developmental abnormalities. For instance, pregnant women may not accurately report the extent of their drinking [10], and maternal alcohol use is often concurrent with other factors that pose risk to the developing fetus such as smoking, illicit drug use, poor nutrition, and psychiatric illness [11-13]. Many difficulties inherent in human studies, however, can be overcome by animal models of fetal alcohol exposure [14], which allow for control over the amount and timing of alcohol intake as well as the isolation of drinking from other confounding factors. Furthermore, animal models provide opportunities to investigate the neurobiological mechanisms underlying fetal alcohol effects and to determine if those effects persist across the lifespan.

Here, we used a mouse model to investigate the impact of fetal alcohol exposure on brain development and behavioral outcome. Throughout pregnancy, mouse mothers drank a 10% ethanol (EtOH) solution. When offspring reached adulthood, we used high-resolution magnetic resonance imaging (MRI) to perform a brain-wide screen for structural abnormalities and found that the olfactory bulbs (OB) exhibited the largest reduction in volume following fetal alcohol exposure. Next, we used an associative olfactory task to assess odor discrimination and odor memory and found that adult mice with fetal alcohol exposure displayed impairment in ability to discriminate between odors with a high degree of similarity. Finally, using an *in vitro *neurosphere assay and *in vivo *cell labeling techniques, we found that the reduced OB volume and olfactory deficits in mice with fetal alcohol exposure were associated with disruptions in postnatal OB neurogenesis.

## Results

### Fetal alcohol exposure results in structural brain abnormalities in adult mice

**Figure 1:**
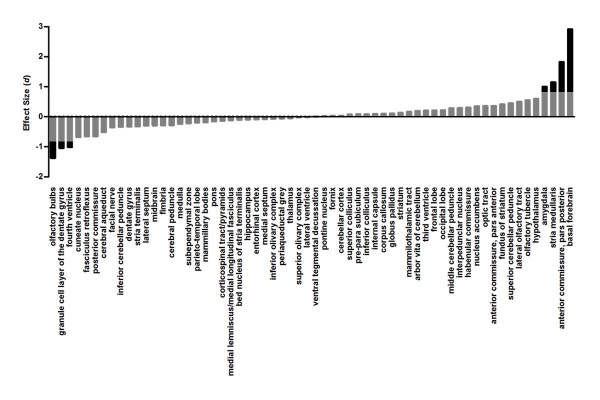
**Effect of fetal alcohol exposure on brain volume**. High-resolution MRI was used to screen for volumetric differences between adult control (*n *= 10) and FAE (*n *= 10) mice in 62 brain regions. Negative and positive effect sizes indicate fetal alcohol exposure-induced decreases and increases in volume, respectively, with an effect size of ≥ 0.8 or ≤ -0.8 considered large (black bars).

Children whose mothers consumed alcohol during pregnancy possess structural abnormalities in several brain regions [3-5], many of which are also observed in mouse embryos exposed to alcohol during gestation [15]. To determine whether these structural abnormalities persist into adulthood, we used MRI due to its whole brain coverage and excellent soft tissue contrast to screen for anatomical differences between control and fetal alcohol-exposed (FAE) mice on postnatal day (P) 60. We found that fetal alcohol exposure produced a negligible decrease in total brain volume (*d *= -0.2). After normalizing for total brain size, however, FAE mice exhibited both decreases and increases in the volume of individual brain regions (Figure 1). The regions exhibiting the largest decreases in volume were the olfactory bulbs (OB), the granule cell layer of the dentate gyrus region of the hippocampus, and the fourth ventricle. The regions exhibiting the largest increases in volume were the basal forebrain, the posterior part of the anterior commissure, the stria medullaris of the thalamus, and the amygdala. These findings suggest that structural brain abnormalities resulting from fetal alcohol exposure persist into adulthood and may contribute to long-lasting behavioral alterations.

### Assessment of olfactory behavior using associative olfactory task

**Figure 2:**
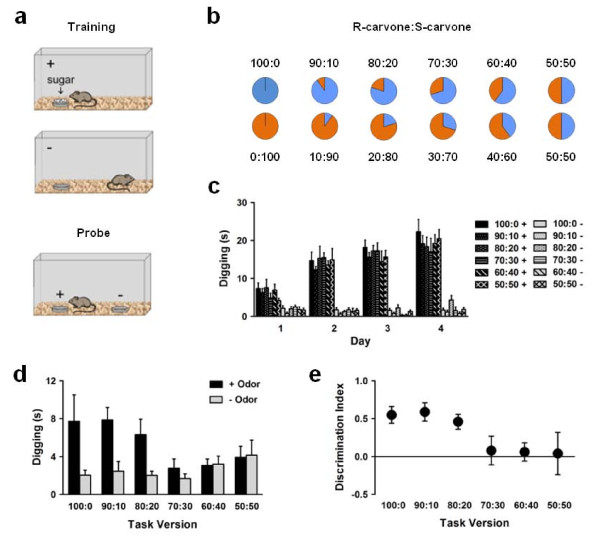
**Associative olfactory task**. (a) Mice were trained to discriminate between two odors using an associative olfactory task in which the + odor but not the -odor was paired with sugar. (b) The + and -odors were composed of increasingly similar binary mixtures of R-carvone and S-carvone. (c) During training, mice spent more time digging at the + odor compared to the -odor regardless of odor similarity (100:0: *n *= 10; 90:10: *n *= 9; 80:20: *n *= 8; 70:30: *n *= 8; 60:40: *n *= 9; 50:50: *n *= 8). (d) During a probe test given 24 hours after training, mice spent more time digging at the + odor compared to the -odor when the odors were relatively distinct (100:0, 90:10, 80:20) but not when the odors were relatively similar (70:30, 60:40) or identical (50:50). (e) Mice had discrimination indices above chance level only when the odors were relatively distinct (100:0, 90:10, 80:20).

Because the OB exhibited the largest reduction in volume, we investigated whether fetal alcohol exposure produces deficits in olfactory behavior during adulthood. Starting at P60, adult mice were trained in an associative olfactory task in which one odor (+ odor) was always paired with a sugar reward and the other odor (-odor) was never paired with sugar (Figure 2a). If mice could discriminate between the + and -odors and selectively associate the + odor with sugar, then they were expected to dig preferentially in the bedding on top of the + odor during a post-training probe test. To vary the difficulty of the discrimination, we used increasingly similar binary mixtures of R-carvone (spearmint) and S-carvone (caraway) as the + and -odors (Figure 2b). In the easiest version of the task (i.e., 100:0), mice were trained to discriminate between an odor composed of 100% R-carvone/0% S-carvone and an odor composed of 0% R-carvone/100% S-carvone. In a more difficult version (i.e., 80:20), mice were trained to discriminate between an odor composed of 80% R-carvone/20% S-carvone and an odor composed of 20% R-carvone/80% S-carvone. To vary memory demands, the delay between training and the probe test was 10 min, 24 hours, or 7 days. Thus, by using a single task and varying two of its parameters, we could assess ability to discriminate between similar odors as well as ability to remember an association between an odor and a reward.

Before testing fetal alcohol-exposed mice, we first assessed how well normal mice could discriminate between odors of increasing similarity. Separate groups of untreated mice were trained in all six versions of the task. During training, mice spent more time digging at the + odor compared to the -odor regardless of task version (Figure 2c) (version × odor × day analysis of variance (ANOVA); odor main effect: *F*(1,59) = 740.14, *p *< .001). Furthermore, the amount of time spent digging at the + odor increased across days, whereas the amount of time spent digging at the -odor remained low (version × odor × day ANOVA; day × odor interaction: *F*(3,177) = 53.30, *p *< .001). Notably, mice spent more time digging at the + odor than the -odor even when the two odor mixtures were identical (50:50), suggesting that it was the presence of sugar and not the ability to discriminate between odors that determined whether mice persisted in digging during training. During the probe test 24 hours later, when no sugar was present, mice dug preferentially at the + odor only in certain versions of the task (Figure 2d) (version × odor ANOVA; version × odor interaction: *F*(5,46) = 2.81, *p *= .027). Specifically, mice in the 100:0, 90:10, and 80:20 versions of the task spent more time digging at the + odor compared to the - odor (100:0: *t*(9) = 2.27, *p *= .049; 90:10: *t*(8) = 4.05, *p *= .004; 80:20: *t*(7) = 2.91, *p *= .023), indicating that they discriminated between odors that were relatively distinct. Mice in the 70:30, 60:40, and 50:50 versions of the task, however, spent equivalent amounts of time digging at the + and -odors, indicating that they did not discriminate between odors that were relatively similar or identical. To further characterize task performance, we computed a discrimination index with '1' denoting digging exclusively at the + odor and '0' denoting equivalent amounts of digging at the + and -odors (i.e., chance level). We found that the discrimination index differed depending on task version (version ANOVA; version main effect: *F*(5,46) = 3.70, *p *= .007). Only mice in the 100:0, 90:10, and 80:20 versions of the task had discrimination indices significantly above chance level (Figure 2e) (100:0: *t*(9) = 5.03, *p *= .001; 90:10: *t*(8) = 4.82, *p *= .001; 80:20: *t*(7) = 4.54, *p *= .003).

### Fetal alcohol exposure impairs odor discrimination in adult mice

**Figure 3:**
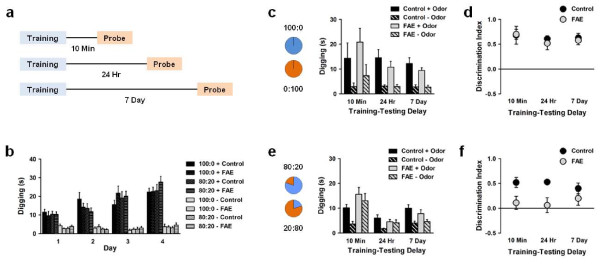
**Effect of fetal alcohol exposure on associative olfactory task performance**. (a) Adult control and FAE mice were trained to discriminate between two relatively distinct odors (100:0) or two relatively similar odors (80:20), with a 10 min (100:0: control *n *= 7, FAE *n *= 8; 80:20: control *n *= 9, FAE *n *= 9), 24 hour (100:0: control *n *= 8, FAE *n *= 8; 80:20: control *n *= 9, FAE *n *= 9), or 7 day (100:0: control *n *= 9, FAE *n *= 9; 80:20: control *n *= 10, FAE *n *= 9) delay between training and testing. (b) Both control and FAE mice spent more time digging at the + odor compared to the -odor during training. (c,d) Both control and FAE mice discriminated between + and -odors when the odors were relatively distinct (100:0 vs. 0:100), regardless of the delay between training and testing. (e,f) FAE mice failed to discriminate between + and -odors when the odors were relatively similar (80:20 vs. 20:80).

To examine whether fetal alcohol exposure impairs ability to discriminate between odors, we trained control and FAE mice in either the 100:0 or 80:20 version of the task. These versions were chosen because they were the easiest and most difficult versions, respectively, that were successfully performed by untreated mice. Also, to examine whether fetal alcohol exposure impairs ability to remember an association between an odor and a reward, we varied the delay between training and the probe test (Figure 3a). During training, mice spent more time digging at the + odor compared to the -odor regardless of task version (Figure 3b) (version × group × odor × day ANOVA; odor main effect: *F*(1,65) = 520.04, *p *< .001), and the amount of time spent digging at the + odor but not the -odor increased across days (version × group × odor × day ANOVA; day × odor interaction: *F*(3,195) = 32.70, *p *< .001). Importantly, we found no differences between groups during training, indicating that both control and FAE mice detected the sugar in the bedding during + trials and had similar levels of motivation to perform the task.

During the probe test for the 100:0 task, we found that both control and FAE mice spent more time digging at the + odor compared to the -odor regardless of the delay between training and testing (Figure 3c) (group × odor × delay ANOVA; odor main effect: *F*(1,42) = 69.04, *p *< .001). Both control and FAE mice had discrimination indices significantly above chance level at all delays (Figure 3d) (control 10 min: *t*(6) = 3.70, *p *= .010; control 24 hours: *t*(7) = 10.91, *p *< .001, control 7 days: *t*(8) = 7.07, *p *< .001; FAE 10 min: *t*(7) = 6.89, *p *< .001, FAE 24 hours: *t*(7) = 3.91, *p *= .006, FAE 7 days: *t*(7) = 5.57, *p *= .001). Therefore, when the + and -odors were relatively distinct, both control and FAE mice discriminated between the odors, selectively associated the + odor with sugar, and remembered the odor-sugar association for at least 7 days.

During the probe test for the 80:20 task, however, we observed differences between control and FAE mice in the amount of time spent digging at the + and -odors (Figure 3e) (group × odor × delay ANOVA; group main effect: *F*(1,49) = 7.40, *p *= .009; group × delay interaction: *F*(2,49) = 8.58, *p *= .001; odor main effect: *F*(1,29) = 17.90, *p *< .001). Control mice exhibited higher discrimination indices than FAE mice regardless of the delay between training and testing (Figure 3f) (group × delay ANOVA; group main effect: *F*(1,49) = 14.00, *p *< .001). Whereas control mice had discrimination indices significantly above chance level at all delays, despite evidence of slight forgetting at the longest delay (10 min: *t*(8) = 5.03, *p *= .001; 24 hours: *t*(8) = 9.69, *p *< .001; 7 days: *t*(9) = 3.70, *p *= .005), FAE mice had discrimination indices not significantly different from chance level at all delays. Therefore, when the + and -odors were relatively similar, FAE mice failed to discriminate between odors. Together, these results suggest that the reduction in OB volume by fetal alcohol exposure is associated with a specific impairment in perceptual discrimination between similar odors, leaving ability to learn and remember associations between an odor and a reward intact.

### Associative olfactory task performance is hippocampal-independent

**Figure 4:**
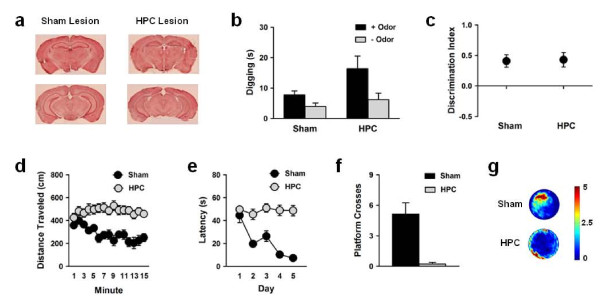
**Associative olfactory task performance does not require the hippocampus**. (a) Representative images of sham and hippocampal (HPC) lesions. (b) Both sham (*n *= 8) and HPC (*n *= 9) mice spent more time digging at the + odor compared to the -odor. (c) Both sham and HPC mice had discrimination indices above chance level. (d) HPC mice did not show habituation of activity levels in the open field. (e,f) HPC mice did not learn the location of a hidden platform in the water maze. (g) Density plots for grouped data showing where mice concentrated their searches for the hidden platform. The color scale represents number of visits.

Because the associative olfactory task involves both perceptual discrimination between odors and the association of an odor with a reward, it might require not only the OB but also other brain regions involved in learning and memory such as the hippocampus. As we observed volumetric reductions in both the OB and the granule cell layer of the dentate gyrus, it is possible that the impairment in performance observed among mice with fetal alcohol exposure could be attributed to abnormal development of the hippocampus rather than the OB. To determine whether performance in the associative olfactory task requires the hippocampus, untreated mice received extensive N-methyl-D-aspartic acid (NMDA) lesions of the hippocampus (Figure 4a) and were trained in the 80:20 version of the task. During the probe test given 24 hours after training, both sham and hippocampal-lesion (HPC) mice spent more time digging at the + odor compared to the -odor (Figure 4b) (group × odor ANOVA; odor main effect: *F*(1,15) = 26.00, *p *< .001), and both sham and HPC mice had discrimination indices significantly above chance level (Figure 4c) (sham: *t*(7) = 4.04, *p *= .005; HPC: *t*(8) = 3.64, *p *= .007), indicating that HPC mice learned to discriminate between two similar odors. Also, we observed that HPC mice spent more time digging at the + odor compared to sham-lesion mice (Figure 4b) (group × odor ANOVA; group × odor interaction: *F*(1,15) = 5.29, *p *= .036), consistent with previous reports of hyperactivity following hippocampal lesions [16,17].

To verify the effectiveness of the lesions, we next tested HPC mice in the open field and the water maze. As expected based on previous studies [18,19], HPC mice showed no habituation of activity levels in the open field (Figure 4d) (group × minute ANOVA; group × minute interaction: *F*(14,210) = 5.49, *p *< .001), no improvement in latency to find a hidden platform across water maze training (Figure 4e) (group × day ANOVA; group × day interaction: *F*(4,60) = 6.89, *p *< .001), and no evidence of a spatial bias for the platform location after water maze training (Figure 4fg) (group ANOVA; group main effect: *F*(1,15) = 21.68, *p *< .001), thereby minimizing the possibility that successful performance in the associative olfactory task was supported by residual hippocampal tissue. The finding that the hippocampus is not necessary for associative olfactory task performance, together with the observation that other olfactory areas of the brain (e.g., olfactory tubercule, lateral olfactory tract) show relatively small changes in volume following fetal alcohol exposure, strengthens the likelihood that the impairment in odor discrimination observed among FAE mice is due to abnormal development of the OB as opposed to other brain regions.

### Fetal alcohol exposure reduces OB neurogenesis during early postnatal development

As the OB is one of the brain regions to which new neurons continue to be added after birth and into adulthood [20,21], we next investigated whether deficits in postnatal neurogenesis may contribute to the reduced OB volume and impaired odor discrimination among FAE mice.

**Figure 5:**
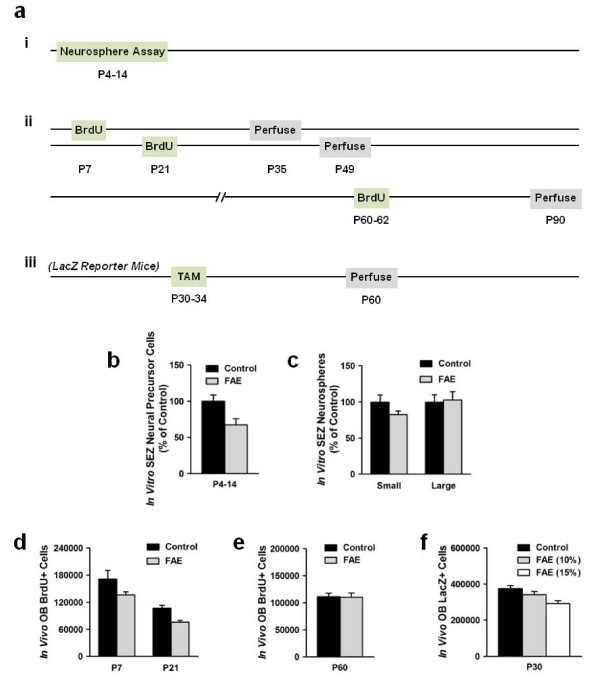
**Effect of fetal alcohol exposure on olfactory bulb neurogenesis**. (a) Postnatal OB neurogenesis was assessed using 3 methods: (i) *in vitro *neurosphere assay, (ii) *in vivo *labeling with BrdU, and (iii) *in vivo *labeling with LacZ using a transgenic reporter system. (b,c) FAE mice (*n *= 9) had fewer *in vitro *neural precursor cells and fewer small neurospheres in the SEZ compared to control mice (*n *= 9) between P4 and P14. (d) FAE mice had fewer BrdU+ cells in the granule cell layer of the OB compared to control mice P7 (control *n *= 9, FAE *n *= 8) and P21 (control *n *= 8, FAE *n *= 9). (e) There was no difference in BrdU+ cells between control (*n *= 8) and FAE mice (*n *= 8) at P60. (f) FAE reporter mice had fewer LacZ+ cells in the granule cell layer of the OB at P30 (control *n *= 8, FAE (10%) *n *= 6, FAE (15%) *n *= 9).

Postnatally-generated OB neurons originate in the subependymal zone (SEZ) of the lateral ventricles before migrating to the OB via the rostral migratory stream [22]. To examine whether fetal alcohol exposure reduces the pool of neural precursor cells in the SEZ, we performed an *in vitro *neurosphere assay. SEZ tissue was extracted from mice between P4 and P14, dissociated into single cells, and cultured under proliferative conditions for 7 days (Figure 5a). Through bulk culturing, we found that FAE mice had fewer neural precursor cells compared to age-matched control mice (Figure 5b) (*t*(8) = 3.89, *p *= .005). Furthermore, culturing at clonal density demonstrated that FAE mice had fewer small (50-100 μm) neurospheres (*t*(8) = 3.54, *p *= .008) but similar numbers of large (> 100 μm) neurospheres compared to control mice (Figure 5c). Given that small neurospheres are believed to arise from neural progenitor cells with finite capacity for mitotic cell divisions, whereas large neurospheres are believed to arise from neural stem cells with unlimited capacity for self-renewal [23], these findings suggest that fetal alcohol exposure specifically reduces the number of SEZ neural progenitor cells.

A reduction in the number of SEZ neural progenitor cells could lead to a decrease in the number of new neurons arriving at the OB. To examine whether fetal alcohol exposure reduces the number of new OB neurons, we performed *in vivo *labeling of new cells with the nucleoside analog 5-bromo-2'-deoxyuridine (BrdU) (Figure 5a). Mice received a single injection of BrdU at P7 or P21 and were perfused 4 weeks later. Using stereological techniques, we quantified the number of BrdU+ cells in the granule cell layer of the OB, the destination of nearly all postnatally-generated OB neurons [24]. We found that the number of BrdU+ cells declined with age for both control and FAE mice (Figure 5d) (group × age ANOVA; age main effect: *F*(1,30) = 172.03, *p *< .001). However, FAE mice had fewer BrdU+ cells than control mice across both ages (group × age ANOVA; group main effect: *F*(1,30) = 10.45, *p *= .003; P7: (*t*(15) = 2.37, *p *= .031; P21: *t*(15) = 2.63, *p *= .019), indicating that fetal alcohol exposure reduces the number of new cells in the OB during the early postnatal period. To determine whether the decrease in OB neurogenesis persists into adulthood, mice received multiple BrdU injections on P60-62 and were perfused 4 weeks later. At this age, we found no difference between control and FAE mice in the number of BrdU+ cells (Figure 5e), suggesting that the effect of fetal alcohol exposure on OB neurogenesis is transient. Together, these findings indicate that deficits in early postnatal OB neurogenesis may underlie the reduction in OB volume and impairment in odor discrimination observed among adult mice with fetal alcohol exposure.

As an additional test of whether fetal alcohol exposure reduces the number of new OB neurons, we performed *in vivo *labeling of new cells using a genetic reporter system (Figure 5a). NestinCreER^T2 ^× RosaLacZ transgenic mice were injected with tamoxifen (TAM) on P30-34, leading to expression of the LacZ transgene in nestin+ cells [25,26], and were perfused 4 weeks later. We found that FAE mice with mothers that drank 10% EtOH during pregnancy exhibited a small but non-significant decrease in the number of LacZ+ cells in the granule cell layer of the OB compared to control mice (Figure 5f). However, mice with mothers that drank 15% EtOH exhibited a larger, significant decrease in the number of LacZ+ cells (group ANOVA; group main effect: *F*(2,20) = 6.18, *p *= .008; 15% vs. control: *t*(15) = 3.42, *p *= .004). These findings suggest that the magnitude of the deficit in postnatal OB neurogenesis following fetal alcohol exposure depends on the amount of alcohol consumed during pregnancy.

## Discussion

Using a mouse model, we aimed to establish a connection between fetal alcohol-induced changes in brain development and alterations in behavioral outcome by first identifying the structural brain abnormalities that persist into adulthood and then linking those brain abnormalities with specific modifications in behavior. Using high-resolution MRI, we observed both increases and decreases in the size of particular brain regions, including the basal forebrain, anterior commissure, amygdala, and the granule cell layer of the dentate gyrus. Amid these widespread changes in brain volume, however, the largest reduction in volume occurred in the OB. We found that this decrease in OB volume during adulthood may arise from deficits in OB neurogenesis occurring specifically during early development, as mice with fetal alcohol exposure possessed fewer neural progenitor cells in the SEZ and fewer new cells in the granule cell layer of the OB during the first few postnatal weeks, but normal numbers of new cells in the OB during adulthood. To determine the long-term consequences of abnormal OB development for olfactory behavior, we tested adult mice in an associative olfactory task designed to assess both odor discrimination and odor memory. We found that although fetal alcohol-exposed mice learned and remembered an association between an odor and a reward, they failed to discriminate between odors with a high degree of similarity. Therefore, using a combination of techniques, including structural brain imaging, *in vitro *and *in vivo *cell detection methods, and behavioral testing, we found that fetal alcohol exposure affects the initial formation of the OB and produces deficits in olfactory behavior that are evident during adulthood. These findings demonstrate that exposure to alcohol during gestation can have a significant impact on brain development, thereby leading to disturbances in behavior that persist into adulthood.

### Fetal alcohol exposure produces abnormalities in OB development in rodents

Our finding of reduced OB volume in fetal alcohol-exposed mice is consistent with previous studies examining the effect of alcohol on brain development. Gross observations [27] and MRI studies [28,29] of mouse embryos show that the OB is one of the brain regions that is reduced in volume following fetal alcohol exposure. Also, studies estimating volume from serial brain sections show that exposure to alcohol during gestation (equivalent to the 1^st ^and 2^nd ^trimesters in humans) or the first ten postnatal days (equivalent to the 3^rd ^trimester in humans) reduces OB volume in pups [30-33] and adult rodents [34]. In the present study, we performed a brain-wide MRI screen for structural changes in adult mice with fetal alcohol exposure and found that out of 62 brain regions, the OB was the region exhibiting the largest reduction in volume. Thus, whereas most previous studies observed reductions in OB volume shortly after alcohol exposure (i.e., in prenatal or early postnatal rodents), we found that this fetal alcohol-induced reduction in OB volume is not only long-lasting, but is also one of the most prominent structural changes evident during adulthood. Taken together, therefore, these studies indicate that the OB is a brain region that is particularly vulnerable to fetal alcohol exposure, with alcohol-induced structural abnormalities emerging early in development and persisting into adulthood.

This reduction in OB volume could result from multiple contributing mechanisms, including an increase in cell death or a decrease in the generation of new cells. Here, we explored whether fetal alcohol exposure decreases the addition of new cells to the granule cell layer of the OB. Granule cells are the most populous cell type in the OB [35] and are predominantly generated during the first few weeks of postnatal life [36]. We found that young mice with fetal alcohol exposure possessed fewer newly-generated granule cells in the OB, which agrees with previous studies showing that pups with fetal alcohol exposure have fewer granule cells in the OB [30] and a disproportionate decrease in the volume of the granule cell layer compared to other cell layers in the OB [37]. Also, in accord with a previous report that fetal alcohol exposure reduces the number of neural stem cells in the SEZ [38], we found that young mice with fetal alcohol exposure possessed fewer SEZ neural progenitor cells, suggesting that the decrease in developmentally-generated OB granule cells results from a deficiency in SEZ cell proliferation. Consistent with a change in the number of granule cells, which are inhibitory neurons that synthesize brain-derived neurotrophic factor (BDNF) [39,40], young rodents with fetal alcohol exposure also exhibited alterations in GABA levels [41], GABA-related gene expression [42], and BDNF levels in the OB [33]. Therefore, a decrease in the number of granule cells may lead not only to a reduction in overall OB volume but also to alterations in neurotransmission and neurotrophin expression within the OB.

### Fetal alcohol exposure produces disturbances in olfactory behavior in rodents

The observation of fetal alcohol-induced abnormalities in OB development raises the question of whether rodents with fetal alcohol exposure display disturbances in olfactory-related behavior. Previous studies indicate that rodents exposed to alcohol during gestation display a preference for the odor of ethanol later in life, particularly during adolescence [43,44]. Furthermore, fetal alcohol exposure has been observed to result in impairments in nonassociative and associative odor learning in very young rodents. Specifically, compared to control pups, alcohol-exposed pups displayed less habituation of a cardiac response to an odor across repeated presentations [45,46]. Furthermore, fetal alcohol-exposed pups failed to associate an odor with either an appetitive or an aversive stimulus [47]. Adult rodents with fetal alcohol exposure, however, showed normal associative odor learning [47], suggesting that fetal alcohol-induced impairments in odor learning might dissipate with age. In the present study, we used a more sensitive olfactory task that allowed us to assess memory for an odor-reward association as well as perceptual discrimination between increasingly similar odors. We found that, consistent with a previous study [47], adult mice with fetal alcohol exposure displayed normal odor learning, as they were able to associate an odor with a reward and remember this association for at least 7 days. However, they failed to discriminate between odors with a high degree of similarity. Therefore, we found that exposure to alcohol during gestation impairs ability to perceive subtle differences between odors and, furthermore, that this impairment persists into adulthood. Because discrimination between similar odors is facilitated through granule cell-mediated lateral inhibition in the OB [48,49], we speculate that the poor odor discrimination among fetal alcohol-exposed mice could arise from non-optimal levels of lateral inhibition resulting from fewer numbers of developmentally-generated granule cells in the OB.

### Contribution of developmental vs. adult neurogenesis to olfactory behavior

The role of OB neurogenesis in olfactory behavior is currently under debate, with some studies reporting that reduction of neurogenesis impairs odor memory [50-53] and others reporting that reduction of neurogenesis impairs odor discrimination [54-56]. As suggested by Lazarini and colleagues [53], this apparent discrepancy could be explained by the time in development at which OB neurogenesis is reduced. When OB neurogenesis was reduced throughout the lifespan using unconditional genetic mutations, impairments in odor discrimination were observed [54-56]. However, when OB neurogenesis was reduced only during adulthood using irradiation or antimitotic drugs, odor discrimination was preserved, but odor memory was disrupted [50-53]. This suggests that developmentally-generated, but not adult-generated, OB neurons are important for odor discrimination. The present study offers additional support for this hypothesis. Specifically, FAE mice showed deficits in OB neurogenesis during the first few postnatal weeks but normal OB neurogenesis during adulthood, and this selective reduction in developmentally-generated OB neurons was associated with impairment in odor discrimination but not odor memory. Together, therefore, these findings suggest that the initial formation of the OB via the production of new neurons during early development may be critical for the ability to discriminate between odors, whereas the continued addition of new neurons to the OB during adulthood may support odor memory.

### Brain-wide effects of fetal alcohol exposure in rodents

Similar to the widespread changes in brain structure observed among children with FASD [3-5], we found brain-wide changes in the volume of several regions in adult mice with fetal alcohol exposure, including the granule cell layer of the dentate gyrus, basal forebrain, anterior commissure, and amygdala. In particular, our observation of a decrease in the volume of the granule cell layer of the dentate gyrus is consistent with previous, well-documented findings of altered hippocampal structure and function among adult rodents with fetal alcohol exposure [57,58], including lower cell counts [59,60], reduced neurogenesis [61,62], deficits in synaptic plasticity [63,64], and impaired performance in water maze and contextual fear memory tasks [65-69]. Our observation of an increase in the volume of the basal forebrain during adulthood also extends previous findings of abnormal basal forebrain structure [32] and cholinergic neurotransmission [70] in rodent pups following fetal alcohol exposure. Interestingly, cholinergic input to the OB has been found to modulate perceptual discrimination between similar odors [71,72]. Therefore, future studies could investigate whether fetal alcohol exposure-induced alterations in cholinergic transmission from the basal forebrain to the OB might contribute to impairments in odor discrimination.

## Conclusions

Using a mouse model, we found that exposure to alcohol during gestation results in smaller OBs and impairments in odor discrimination that persist into adulthood. Furthermore, we found that these abnormalities in OB structure and function may arise from deficits in the generation of new OB neurons from the SEZ during the first few postnatal weeks. Although controversial [73], there is evidence that, like mice, humans also exhibit postnatal OB neurogenesis, with new cells originating in the SEZ and migrating to the OB [74,75]. Therefore, humans exposed to alcohol during gestation may exhibit changes in OB development similar to those observed in mice. Indeed, autopsies of children with severe FASD revealed anomalies in OB structure [76]. Although it is unknown whether children with FASD exhibit deficits in olfaction, observations that individuals with alcoholism or Korsakoff's syndrome are unable to discriminate between odors [77,78] is suggestive of this possibility. Therefore, our finding of a connection between fetal alcohol-induced abnormalities in OB development and alterations in olfactory behavior provides a model system that can be used to further understand relationships between brain and behavior [79] and points toward new clinical directions in the detection and treatment of FASD.

## Methods

### Mice

All procedures were approved by the Animal Care Committee at The Hospital for Sick Children. Mice were bred in the colony at The Hospital for Sick Children and maintained on a 12 hr light/dark cycle (lights on at 0700 hrs). Control and FAE mice were obtained from a cross between female 129Svev and male C57Bl/6 mice (Taconic). In one experiment, control and FAE mice were obtained from a cross between female wild-type 129Svev and male CreER^T2 ^heterozygous × RosaLacZ homozygous mice in a C57Bl/6 background. Genotypes were determined using PCR analysis of tail DNA samples. After weaning on P21, mice were group-housed (2-5 per cage) in transparent plastic cages (31 × 17 × 14 cm) and allowed free access to food and water unless otherwise specified. To reduce the possibility of litter effects on dependent measures [80], mice in each experimental group were sampled from between 3 and 6 different litters.

### Fetal alcohol exposure

The fetal alcohol exposure procedure was adapted from Allan and colleagues [81-83]. Individually-housed female mice (*n *= 29) were given water containing 10% EtOH + 0.1% saccharin in place of regular drinking water. Before pregnancy, the concentration of EtOH in the water was gradually increased to 10% (2 days at 2%, 2 days at 5%, 7 days at 10%). Mice continued to receive 10% EtOH during a 4-day period of cohabitation with a male breeder and throughout pregnancy. Starting on the day of birth (P0), the concentration of EtOH was gradually decreased (2 days at 5%, 2 days at 2%) and then replaced with regular water. Control mice (*n *= 29) received water containing 0.1% saccharin throughout pregnancy. In the CreER^T2 ^× RosaLacZ experiment, a subset of mice (*n *= 3) received 15% EtOH + 0.1% saccharin, with the concentration of EtOH gradually increased before pregnancy (2 days at 4%, 2 days at 8%, 7 days at 15%) and decreased starting on P0 (2 days at 8%, 2 days at 4%). Control, 10%, and 15% mice consumed an average of 4.14 ± 0.09 g, 3.80 ± 0.07 g, and 3.55 ± 0.05 g (mean ± SEM) fluid each day during pregnancy, resulting in an average daily EtOH dose of 0 ± 0 g/kg, 13.60 ± 0.23 g/kg, and 20.87 ± 1.65 g/kg, respectively. Although mice continued to drink a reduced amount of EtOH during the first few days after giving birth, the amount of EtOH passed on to pups through lactation is low (~2% of maternal dose) [84]. Therefore, it is expected that the amount of EtOH received by pups during this period was minimal.

Consistent with a previous study showing that drinking 10% EtOH does not reduce food consumption [81], we found no differences among groups in body weight at the onset of pregnancy (control: 24.46 ± 0.85 g, 10%: 24.59 ± 0.77 g, 15%: 23.70 ± 2.70 g; group ANOVA, group main effect: *p *= .49) or weight gained across pregnancy (control: 11.68 ± 0.53 g, 10%: 12.61 ± 0.53 g, 15%: 16.13 ± 1.59 g; group ANOVA, group main effect: *p *= .08), although there was a non-significant trend toward greater weight gain in 15% EtOH mice compared to control (Tukey's post-hoc test: *p *= .06) or 10% EtOH mice (Tukey's post-hoc test: *p *= .09). Also, we observed no differences among groups in litter size (control: 6.26 ± 0.38 pups, 10%: 6.32 ± 0.39 pups, 15%: 7.67 ± 0.88 pups; group ANOVA, group main effect: *p *= .54). Furthermore, consistent with a previous study showing that drinking 10% EtOH during pregnancy does not disrupt maternal care behaviors, including licking and grooming of pups and time spent on the nest [81], we observed no differences among groups in pup weight across the pre-weaning period (P3: control: 2.64 ± 0.09 g, 10%: 2.79 ± 0.08 g, 15%: 2.49 ± 0.09 g; P10: control: 7.28 ± 0.23 g, 10%: 7.46 ± 0.24 g, 15%: 7.28 ± 0.29 g; P21: control: 14.14 ± 0.33 g, 10%: 14.38 ± 0.38 g, 15%: 13.90 ± 0.35 g; age × group ANOVA, age × group interaction: *p *= .96, group main effect: *p *= .61, age main effect: *F*(2, 116) = 1060.08, *p *< .001).

This drinking procedure in mice [81,83] (and similar procedures in rats [63,85]) offers a number of advantages over other EtOH administration methods. First, EtOH is delivered through drinking water instead of via gastric intubation or injection, which reduces the amount of stress to mothers or pups. Second, mice have free access to regular food at all times instead of receiving a liquid diet as their sole nutrient source. Third, the steady decrease in EtOH dose during the first few days after birth allows for gradual withdrawal from EtOH without disrupting maternal care-giving behaviors. Therefore, this drinking procedure lessens or eliminates the need for a pair-fed condition or cross-fostering of pups to non-EtOH-consuming dams, while producing changes in offspring brain and behavior that replicate those observed following EtOH-containing liquid diets [64,66,67].

### MRI

MRI methods were similar to those previously described by Lerch et al. [86,87]. On P60, male mice were anaesthetized with choral hydrate and perfused transcardially with phosphate buffered saline (PBS) + 1 μl/ml heparin (Pharmaceutical Partners of Canada) + 2 mM ProHance (Bracco Diagnostics Inc.) followed by 4% paraformaldehyde (PFA) + 2 mM ProHance. Skulls were separated from the bodies, and the skin, lower jaw, ears, and cartilaginous nose tip were removed. The remaining skull structures containing the brain were postfixed overnight in PFA + 2 mM ProHance and stored at 4°C in PBS + 0.02% sodium azide + 2 mM ProHance.

A multi-channel 7.0-T MRI scanner (Varian Inc.) with a 6-cm inner bore diameter insert gradient was used to acquire anatomical images of brains within skulls. Before imaging, skulls were removed from solution, blotted, and placed into plastic tubes (13 mm diameter) filled with a proton-free susceptibility-matching fluid (Fluorinert FC-77, 3 MCorp). Three custom-built solenoid coils (14 mm diameter, 18.3 cm length) with over-wound ends were used to image three brains in parallel. Parameters used in the scans were optimized for grey/white matter contrast: a T2-weighted, 3D fast spin-echo sequence with 6 echoes, with TR/TE = 325/32 ms, four averages, field-of-view 14 × 14 × 25 mm^3^, and matrix size = 432 × 432 × 780 giving an image with 32 μm isotropic voxels. Total imaging time was 11.3 hours. Geometric distortion due to position of the coils inside the magnet was calibrated using a precision machined MR phantom. To assess anatomical differences between brains from control and FAE mice, an image registration-based approach was used to compute volumes for 62 structures from each brain using an anatomical atlas [88].

### Associative olfactory task

Starting at P60, male and female mice were trained and tested in an associative olfactory task adapted from Schellinck et al. [89]. Forty-eight hours before training, mice were placed on food restriction to maintain a 10% reduction in free-feeding body weight. To familiarize mice with the reward used during training, ~20 sugar pellets (sucrose, 10 mg each, TestDiet) were given to each mouse 24 hours before training. Training and testing occurred in transparent plastic cages (31 × 17 × 14 cm). Odors were diluted in mineral oil (1:100), applied to filter paper (50 μl), and placed inside of petri dishes (15 mm diameter) with 10 small holes drilled in the lid. Dishes were placed on the bottom of the cage and covered with bedding. Odors consisted of binary mixtures of the enantiomers R-(-)-carvone (98% purity, Sigma) and S-(+)-carvone (96% purity, Sigma). During reinforced trials, one of the odors (+ odor) was presented with 6 sugar pellets placed under the bedding on top of the dish. During non-reinforced trials, the other odor (- odor) was presented with no sugar pellets Mice received one 5-min reinforced trial and one 5-min non-reinforced trial per day for 4 days, with trial order counterbalanced across days. Within each group of mice, particular odor mixtures served as + and -odors a roughly equal number of times.

Ten min, 24 hours, or 7 days after training, mice received a 10-min probe test during which the + and -odor were presented simultaneously. No sugar pellets were present during the probe test. Mice were maintained on food restriction if the delay between training and testing was 10 min or 24 hours. If the delay between training and testing was 7 days, food restriction was terminated after training, and mice were deprived of food 24 hours before the probe test. Amount of time spent digging at the + and -odors (i.e., moving bedding directly on top of the dishes with snout or forepaws) was recorded with a video camera and coded manually. Discrimination between odors was measured using a discrimination index defined as (digging_+ odor _-digging_-odor_)/(digging_+ odor _+ digging_- odor_).

### Hippocampal lesions

Female mice were treated with atropine (0.1 mg/kg) and anesthetized with chloral hydrate (400 mg/kg). To prevent seizure activity associated with excitotoxic lesions, mice were also pretreated with diazepam (5 mg/kg). Using stereotaxic procedures, NMDA (10 mg/ml) was infused into the following 8 sites (4 per hemisphere) with respect to bregma (posterior, lateral, ventral): -2.0 mm, ± 1.5 mm, 1.8 mm; -2.5 mm, ± 1.8 mm, 2.0 mm; -3.0 mm, ± 2.7 mm, 3.0 mm; and -3.0 mm, ± 2.7 mm, 3.5 mm. A volume of 0.1 μl was infused at the 3 most dorsal sites, and a volume of 0.15 μl was infused at the most ventral site. NMDA was delivered via a 32-gauge microsyringe (Hamilton). A pump maintained the infusion rate at 0.1 μl/min and the syringe was left in place for 5 minutes after each infusion. Sham mice were treated identically except no NMDA was infused. Mice were postoperatively treated with ketoprofen (5 mg/kg).

After 7 days of recovery, mice underwent training and testing in the 80:20 version of the associative olfactory task as previously described. Mice also underwent testing in the open field and the water maze. For the open field, mice were released into the center of a square plastic chamber (45 × 45 × 20 cm) and allowed to explore for 15 min under dim light. Total distance traveled was recorded by an overhead video camera and analyzed using automated software (Actimetrics). For the water maze, a circular plastic pool (120 cm diameter, 50 cm height) was filled to a depth of 40 cm with water maintained at ~26°C. Water was made opaque by the addition of nontoxic paint. A circular escape platform (10 cm diameter) was submerged 0.5 cm below the water surface in a fixed location within the center of one of the pool quadrants. The pool was surrounded by curtains, located at least 1 m from the pool wall, that were painted with distinct geometric cues. Mice received 3 trials per day for 5 days. Trials started when mice were released into the pool, facing the wall, from one of 4 possible points. A different release point was used for each trial on each day, and the order of release points varied pseudorandomly across days such that each point was used a roughly equal number of times. Trials ended when mice reached the platform or 60 s elapsed. If a mouse failed to find the platform, it was guided by the experimenter. The inter-trial interval was ~10 min. After the final training trial, mice received a 60-s probe test with the platform removed from the pool. Swim paths were recorded by an overhead video camera. Latency to reach the platform during training and number of platform crosses during the probe trial was analyzed using Actimetrics software. Density plots were generated using custom software created in our laboratory.

After behavioral testing, mice were anesthetized with chloral hydrate and perfused transcardially with PBS followed by 4% PFA. Brains were postfixed in 4% PFA overnight, transferred to 30% sucrose, and stored at 4°C. Coronal sections (50 μm) across the entire anterior-posterior extent of the hippocampus were cut using a cryostat. Sections were mounted on gelatin-coated slides, stained with neutral red, and cover-slipped with Cytoseal. Using Stereo Investigator software, the entire hippocampus and the area of the hippocampus sustaining damage (i.e., cell loss or absence of tissue) were outlined separately for every fourth section. The proportion of total hippocampal tissue damaged by NMDA infusion was 67.90 ± 4.90%. Spared tissue was mainly restricted to the most posterior and ventral regions of the hippocampus, with the overlying neocortex exhibiting little or no damage.

### Neurosphere assay

An *in vitro *neurosphere assay [90] was performed on SEZ tissue extracted from male and female mice between P4 and P14. Tissue underwent enzymatic digestion (1.33 mg/ml trypsin + 0.67 mg/ml hyaluronidase + 0.13 mg/ml kynurenic acid) at 37°C for 50 min and was isolated in serum-free media (SFM) with trypsin inhibitor. The tissue was mechanically dissociated into a single cell suspension, and cell density was determined using trypan blue exclusion. SEZ cells were cultured in bulk and at clonal density using SFM containing 20 ng/ml epidermal growth factor (mouse submaxillary, Sigma) + 10 ng/ml fibroblast growth factor-2 (human recombinant, Sigma) + 2 μg/ml heparin (Sigma). For the bulk culture, 2.2 × 10^5 ^cells per mouse were cultured in 25 ml of SFM. After 7 days, the total number of cells was calculated from the cell density, which was determined using trypan blue exclusion following mechanical dissociation into a single-cell suspension. For the clonal density culture, 2 cells/μl were plated for each mouse in 24-well uncoated plates (0.5 ml SFM per well). After 7 days, the total number of small (50-100 μm diameter) and large (> 100 μm diameter) spheres formed in each well was counted.

### BrdU and LacZ labeling and quantification

At P7 or P21, male and female mice received a single injection of BrdU (50 mg/kg) dissolved in PBS. At P60, mice received 2 injections of BrdU per day (100 mg/kg, 5 hours apart) for 3 days. Four weeks later, mice were anesthetized with chloral hydrate and perfused transcardially with PBS followed by 4% PFA. Brains were postfixed overnight in PFA, transferred to PBS + 0.01% sodium azide, and stored at 4°C. Coronal sections (40 μm) were cut from the anterior tip of the main OB until the intrusion of the accessory OB. Sections were treated with 1 N HCl at 45°C for 30 min, 1% H_2_0_2 _at room temperature for 15 min, and 0.2 M glycine in PBS at room temperature for 10 min. Sections were incubated with the primary antibody (rat anti-BrdU monoclonal, 1:1000, Accurate Chemicals) at room temperature overnight and the secondary antibody (Alexa-488 goat anti-rat, 1:1000, Molecular Probes) at room temperature for 2 hrs. Antibodies were diluted in blocking solution containing 2.5% bovine serum albumin + 5% goat serum + 0.3% Triton X-100 dissolved in PBS. Sections were counterstained with 4',6-diamidino-2-phenylindole (DAPI, 1:10000, Sigma) and mounted on slides with Permafluor anti-fade medium.

At P30, double transgenic Nestin-CreER^T2 ^× RosaLacZ mice received 1 injection of TAM (180 mg/kg, Sigma) dissolved in 10% EtOH/90% sunflower seed oil per day for 5 consecutive days. Mice were perfused 4 weeks later. OB tissue was prepared as previously described. Sections were treated with 1% SDS for 10 min, 1% H_2_0_2 _for 15 min, and 0.2 M glycine in PBS for 10 min. Sections were incubated with the primary antibody (rabbit polyclonal anti-LacZ, 1:250, Molecular Probes) at room temperature overnight and the secondary antibody (Alexa-488 goat anti-rabbit, 1:500, Molecular Probes) at room temperature for 2 hrs. Sections were counterstained with 4',6-diamidino-2-phenylindole (DAPI, 1:10000, Sigma) and mounted on slides with Permafluor anti-fade medium.

BrdU and LacZ quantification was performed using an Olympus BX61 epifluorescent microscope and Stereo Investigator software. The total number of BrdU+ or LacZ+ cells in the granule cell layer of the OB was estimated using optical fractionator. With a 60 × objective, BrdU+ or LacZ+ cells were counted from every sixth section within 60 μm × 60 μm counting frames equally spaced across a 300 μm × 300 μm grid. Cells that fell along the leftmost and bottommost edges of the counting frame were excluded.

### Statistical analysis

For MRI data, we compared differences between control and FAE mice across all 62 brain regions using a measure of effect size, Cohen's *d*, for which values ≥ 0.8 or ≤ -0.8 indicate a large effect size [91]. For neurosphere assay data, values for FAE mice were expressed as the percentage of values for control mice, and differences between groups were tested using one-sample *t*-tests. For all other data, ANOVAs were performed and followed by unpaired or one-sample *t*-tests. In the experiments in which both males and females were used (associative olfactory task testing, BrdU labeling, and LacZ labeling), we initially included sex as a factor in the ANOVAs. As we found no significant effects involving sex, however, this factor was dropped from analysis.

## Competing interests

The authors declare that they have no competing interests.

## Authors' contributions

KGA, SAK, DV, and PWF conceived of and designed the experiments. KGA carried out the EtOH treatment, ran the behavioral tests, performed surgeries, conducted immunohistochemistry, and analyzed the data. SAK and LC performed the neurosphere assay. ATL participated in EtOH treatment and immunohistochemistry. JPL coordinated the MRI scans and analyzed the MRI data. KGA and PWF wrote the manuscript. All authors read and approved the final manuscript.
